# A regulação assistencial da saúde suplementar no Brasil entre 2000 e 2018

**DOI:** 10.1590/0102-311XPT023524

**Published:** 2025-02-07

**Authors:** Ivo Aurelio Lima, Cristiani Vieira Machado, Sheyla Maria Lemos Lima

**Affiliations:** 1 Universidade Federal do Ceará, Sobral, Brasil.; 2 Escola Nacional de Saúde Pública Sergio Arouca, Fundação Oswaldo Cruz, Rio de Janeiro, Brasil.

**Keywords:** Saúde Suplementar, Regulação e Fiscalização em Saúde, Atenção à Saúde, Supplemental Health, Health Care Coordination and Monitoring, Delivery of Health Care, Salud Complementaria, Regulación y Fiscalización en Salud, Atención a la Salud

## Abstract

Este artigo analisa a atuação da Agência Nacional de Saúde Suplementar (ANS) na regulação assistencial do setor entre 2000 e 2018. Por meio de análise documental, caracterizaram-se as principais iniciativas dessa dimensão regulatória nos cinco mandatos da diretoria da ANS no período. Ademais, o estudo explora as normas legais e infralegais relacionadas ao tema e as principais ações da ANS para a garantia das coberturas e a qualificação da assistência ofertada por operadoras e prestadores de serviços. Essas normativas foram organizadas em três eixos de regulação assistencial: rol de procedimentos e eventos em saúde e cobertura assistencial; segmentação assistencial; e iniciativas de promoção à saúde, prevenção de doenças e qualificação da assistência. Observa-se que as medidas de regulação assistencial implementadas seguiram uma tendência de continuidade, em que pese a variação de orientações políticas e trajetórias profissionais de seus diretores-presidentes ou a configuração da diretoria colegiada. Isso pode ser atribuído a fatores como a profissionalização da regulação e o estabelecimento do arcabouço institucional da ANS. Apesar do caráter microrregulatório da atuação da agência, a regulação assistencial contribuiu para a promoção da atenção integral aos beneficiários de planos de saúde e para a indução de melhores práticas assistenciais pelas operadoras e seus prestadores de serviços. Porém, persistem pressões do empresariado para a flexibilização da regulação assistencial.

## Abstract (en)

This article analyzes the performance of the Brazilian National Regulatory for Private Health Insurance and Plans Agency (ANS, acronym in Portuguese) in the regulation of care in the sector from 2000 to 2018. After documentary analysis, the main initiatives of the regulatory dimension were characterized in the five terms of the agency’s board of directors in the period. Furthermore, the study explores the legal and infra-legal norms related to the theme and the ANS main actions to guarantee coverage and qualification of the assistance offered by operators and service providers. These regulations were organized into three axes of care regulation: list of procedures and events in healthcare and care coverage; care segmentation; and Initiatives for health promotion, disease prevention, and qualification of care. We observed that the measures of care regulation implemented followed a continuity trend, despite the variation in political orientations and professional trajectories of their CEOs or the configuration of the collegiate board. This can be attributed to factors such as the professionalization of the regulation and the establishment of ANS institutional framework. Despite the micro-regulatory nature of the agency’s performance, care regulation contributed to the promotion of comprehensive care for health plan beneficiaries and to the induction of better care practices by operators and their service providers. However, companies stakeholders still pressure public managers, aiming at making care regulation more flexible.

## Abstract (es)

El artículo analiza la actuación de la Agencia Nacional de Salud Suplementaria (ANS, por sus siglas en portugués) en la regulación asistencial del sector entre el 2000 y el 2018. Por medio del análisis documental, se caracterizaron las principales iniciativas de esta dimensión regulatoria en los cinco mandatos de la dirección de la agencia durante el período. Además, el estudio explora las normas legales e infralegales relacionadas con el tema y las principales acciones de la ANS para garantizar la cobertura y la cualificación de la asistencia ofrecida por los operadores y prestadores de servicios. Estas normativas se organizaron en tres ejes de regulación asistencial: relación de procedimientos y eventos en salud y cobertura asistencial; segmentación asistencial; e iniciativas de promoción de la salud, prevención de enfermedades y cualificación de la asistencia. Se observa que las medidas de regulación asistencial implementadas siguieron una tendencia de continuidad, a pesar de la variación en las orientaciones políticas y trayectorias profesionales de sus directores presidentes o configuración de la dirección colegiada. Esto puede atribuirse a factores como la profesionalización de la regulación y el establecimiento del marco institucional de la ANS. A pesar del carácter microrregulador de la actuación de la agencia, la regulación asistencial contribuyó a la promoción de la atención integral a los beneficiarios de planes de salud y a la inducción de mejores prácticas asistenciales por parte de los operadores y sus prestadores de servicios. Sin embargo, persisten presiones del empresariado para flexibilizar la regulación asistencial.

## Introdução

Nas últimas décadas, houve transformações nas relações entre Estado, mercados e a sociedade, que em parte afastaram os sistemas de saúde dos três modelos clássicos: sistema nacional de saúde, seguro social e liberal ^1^ ^,^ ^2^ ^,^ ^3^. Quase todos os sistemas apresentam elementos privados e estatais combinados ao longo de sua construção histórica e social ^4^.

Para melhor compreensão sobre a inserção dos seguros privados nos sistemas de saúde, a Organização para a Cooperação e Desenvolvimento Econômico (OCDE) desenvolveu um modelo de classificação que os conceitua em suplementar, substitutivo, complementar ou primário, conforme a cobertura de serviços. O seguro privado do tipo suplementar é caracterizado pela duplicação de cobertura em relação aos serviços oferecidos por sistemas públicos universais ou seguros sociais compulsórios e está presente em países como Portugal, Espanha, Reino Unido e Itália ^5^ ^,^ ^6^.

Na América Latina, predominam sistemas de saúde segmentados que convivem com sistemas de seguro social, baseados em contribuições trabalhistas. Fogem à regra Brasil e Cuba, que têm sistemas universais de saúde ^7^. Porém, no Brasil, o sistema público cronicamente subfinanciado coexiste e interage com um mercado dinâmico de planos e seguros de saúde privados, que o antecede e se desenvolveu desde os anos 1960 subsidiado pelo Estado, entretanto, sob escassa regulação estatal ^1^ ^,^ ^2^ ^,^ ^3^.

Na década de 1990, em meio ao descontentamento de entidades médicas e de defesa dos consumidores com as limitações de procedimentos e exclusões de coberturas e às resistências do empresariado em defesa do livre mercado no setor, a regulação da saúde suplementar ganhou espaço no debate público e no Congresso Nacional. Desencadeou-se o processo legislativo que culminou na aprovação da *Lei nº 9.656* ^8^, de 3 de junho de 1998. Nela, foi instituída a obrigatoriedade de cobertura para todas as doenças previstas na 10ª Classificação Internacional de Doenças (CID-10), o plano de referência, a proibição da exclusão de beneficiários por doenças ou lesões preexistentes, a proibição da limitação quantitativa ou qualitativa de procedimentos ou tempo de internação ^8^.

A Agência Nacional de Saúde Suplementar (ANS), criada pela *Lei nº 9.961* ^9^, de 28 de janeiro de 2000, é parte do quadro normativo e institucional decorrente das reformas empreendidas no Estado brasileiro durante a década de 1990. Essas mudanças ocorreram em um contexto mundial de fortalecimento do neoliberalismo, que transformou profundamente a atuação estatal, o conjunto das relações sociais e as subjetividades ^10^.

Vinculada ao Ministério da Saúde, a ANS é a autarquia especial responsável pela regulação, pela normatização, pelo controle e pela fiscalização das operadoras de planos de saúde e de suas relações com prestadores de serviços e consumidores ^9^. A agência é gerida por uma Diretoria Colegiada (Dicol) com cinco componentes, um deles assumindo, simultaneamente, as funções de diretor-presidente da agência e da Câmara de Saúde Suplementar.

Outros elementos da estrutura organizacional da ANS são as cinco diretorias que abrangem distintas dimensões do processo regulatório: Diretoria de Normas e Habilitação de Produtos (Dipro), Diretoria de Normas e Habilitação das Operadoras (Diope), Diretoria de Fiscalização (Difis), Diretoria de Desenvolvimento Setorial (Dides) e Diretoria de Gestão (Diges) ^11^. A composição dessas diretorias reflete a acomodação de interesses dos atores do setor e as dinâmicas políticas de suas disputas.

A Dipro é a responsável pela regulação assistencial, isto é, pelo planejamento, pela coordenação, pela organização e pelo controle das atividades de regulação, habilitação e acompanhamento dos planos de saúde comercializados. A Diope responde pela regulação econômico-financeira do setor. As duas são consideradas as principais diretorias da agência devido à sua atuação na edição de normas sobre os produtos ofertados e as operadoras ^11^.

A Diges normatiza processos internos à ANS, sendo responsável pela implementação de políticas, gestão e aprimoramento da agência. A Dides executa ações para a qualificação da assistência à saúde produzida pelos prestadores de serviços, além de monitorar suas relações com as operadoras e o Sistema Único de Saúde (SUS). A Difis fiscaliza a atuação das operadoras e o cumprimento das normas vigentes ^11^.

O marco regulatório da saúde suplementar gerou avanços, como a maior estabilidade das empresas, alcançada pela definição de regras mais rígidas para entrada, permanência e saída do mercado. Medidas como o rol de procedimentos e eventos em saúde, a definição da segmentação assistencial e a qualificação dos prestadores de serviços conferiram maior transparência e proteção aos beneficiários de planos ^1^.

Contudo, conflitos entre os atores do setor quanto à regulação assistencial, refletidos no elevado número de reclamações de beneficiários e na crescente judicialização, indicam lacunas no processo regulatório e limites para a atuação da ANS que não foram superados por essas iniciativas ^12^.

Este artigo objetiva analisar as características da regulação assistencial realizada pela ANS no período entre 2000 e 2018. O foco na dimensão assistencial se justifica por ser a mais próxima dos usuários e por expressar o contexto, as contradições e as disputas políticas historicamente existentes entre os sujeitos envolvidos no setor saúde no Brasil ^13^.

## Metodologia

Este estudo utilizou as contribuições teóricas da análise das políticas de saúde, na perspectiva do institucionalismo histórico, para sistematizar o conjunto complexo de inter-relações de que resulta a política de regulação assistencial da saúde suplementar e demonstrar sua dinâmica de mudanças e continuidades ^14^. Assim, elaborou-se uma estrutura analítica que contemplou o contexto, a institucionalidade e os atos normativos produzidos sobre o tema.

A trajetória da regulação estatal dos planos de saúde pela ANS foi dividida em cinco momentos correspondentes aos mandatos dos seus diretores-presidentes entre 2000 e 2018. Os eixos analíticos foram o contexto, a agenda regulatória e a evolução dos componentes estratégicos para regulação assistencial (rol de procedimentos e eventos em saúde e coberturas assistenciais; segmentação assistencial; e iniciativas de promoção à saúde, prevenção das doenças e qualificação da assistência prestada).

As técnicas de pesquisa foram a análise documental e de dados secundários. Analisaram-se documentos oficiais de domínio público e irrestrito produzidos pelo Ministério da Saúde e pela ANS, além de leis, resoluções do Conselho Nacional de Saúde Suplementar (CONSU), relatórios anuais de gestão da ANS, Resoluções da Diretoria Colegiada (RDC) e Resoluções Normativas (RN) produzidas no período (Tabela 1).

**Tabela 1:** Documentos analisados no processo da pesquisa.

Tipo de documento	n	%
Resolução CONSU	22	4,1
Resolução da Diretoria Colegiada (RDC)	59	11,0
Resolução Normativa (RN)	436	81,3
Relatório anual de gestão da ANS	19	3,5
Total	536	100,0

ANS: Agência Nacional de Saúde Suplementar; CONSU: Conselho Nacional de Saúde Suplementar.

Fonte: elaboração própria.

Os dados secundários relativos ao Índice de Desempenho da Saúde Suplementar (IDSS) foram utilizados para caracterizar a performance assistencial das operadoras em relação ao seu porte para o ano de 2017. Este estudo foi submetido ao Comitê de Ética em Pesquisa da Escola Nacional de Saúde Pública Sergio Arouca, Fundação Oswaldo Cruz (ENSP/FIOCRUZ), em que obteve aprovação por meio do parecer nº 3.805.938 em janeiro de 2020.

## Resultados e discussão

### Momentos da regulação assistencial da saúde suplementar segundo gestões da ANS

#### 2000-2003: estruturação da agência e as bases para regulação assistencial

A ANS foi criada em 2000, durante o segundo governo de Fernando Henrique Cardoso, em um cenário de disputa pelo direcionamento do modelo regulatório entre os Ministérios da Fazenda e da Saúde, vencida pelo último. Foi reflexo disso a autonomia do Ministro José Serra para compor uma Diretoria Colegiada que contemplou gestores governamentais e uma representante dos interesses dos consumidores, em um arranjo que lhe garantiu ascendência sobre a agência ^12^.

O primeiro diretor-presidente da ANS foi o ex-dirigente da Agência Nacional de Vigilância Sanitária (Anvisa) Januário Montone, responsável pela organização interna da agência e operacionalização inicial da regulamentação instituída pela *Lei nº 9.656* ^8^. Esse período foi marcado pela ênfase na regulação econômica, com implementação de dispositivos para garantias financeiras inspirados nos do setor de seguros, visando evitar situações de desequilíbrio financeiro de operadoras que comprometessem a assistência aos beneficiários.

Antes da ANS, a regulação do rol de procedimentos e coberturas assistenciais foi realizada pelo CONSU por meio da RN 10 ^15^ de 1998. Nela, instituiu-se que a inclusão de novos procedimentos ao rol dependeria de proposição pelo Ministério da Saúde e aprovação pelo supracitado conselho ^15^. A partir de sua criação, as atualizações passaram a ser feitas por meio de RN.

Nesse período, instituíram-se normas sobre o fornecimento de informações para o cadastro de beneficiários (RDC 03) e o Sistema de Informações de Produtos (SIP) (RDC 85), fundamentais para o acompanhamento da assistência prestada aos consumidores e que podem ser considerados estruturantes do modelo de regulação assistencial para o setor.

#### 2004-2010: a regulamentação do modelo assistencial e o papel dos sanitaristas na ANS

A chegada do Partido dos Trabalhadores (PT) e de sua coalizão ao governo federal promoveu mudanças na maioria das diretorias da ANS. O PT foi o responsável por três delas, incluindo a indicação do médico sanitarista Fausto dos Santos para a presidência da ANS. O Deputado Roberto Jefferson, do Partido Trabalhista Brasileiro (PTB), indicou o médico Alfredo Cardoso para a Diope. Esse diretor tinha vínculo anterior com a Amil e foi um dos nomes recomendados pelo seu proprietário para componentes da base aliada do governo. Desde então, essa diretoria se tornou um espaço privilegiado de interlocução com o empresariado do setor ^12^.

Em 2007, três diretores foram reconduzidos aos seus cargos, entre eles Fausto dos Santos. As mudanças ocorreram na Difis e Diges, que passaram a ser ocupadas respectivamente por Eduardo Sales, ex-procurador geral da ANS, e Hésio Cordeiro, médico sanitarista vinculado ao movimento sanitário brasileiro ^12^.

Nesse período, foram realizados dois concursos públicos para a ANS, fundamentais para a profissionalização da regulação da saúde suplementar e a estruturação de um modelo regulatório mais estável. No primeiro, foram incorporados à agência 310 servidores e, no segundo, mais 121 especialistas em regulação e analistas administrativos ^16^. Os concursos públicos para a agência contemplaram conteúdos sobre a Saúde Coletiva e o Sistema Único de Saúde (SUS), e foram ofertados aos servidores cursos introdutórios e mestrados em Saúde Coletiva, em parceria com a ENSP/FIOCRUZ e o Instituto de Medicina Social da Universidade Estadual do Rio de Janeiro.

O fortalecimento dos sanitaristas na ANS, demonstrado pela composição da diretoria colegiada, gerou avanços importantes para a regulação assistencial. Naquele período, houve a implementação de um conjunto de medidas indutoras que buscavam construir um modelo de atenção integral à saúde no setor e qualificar a assistência prestada.

A ideia era a de que as operadoras estimulassem os prestadores de serviços a se tornarem produtores de cuidado e os beneficiários a utilizarem os serviços de maneira racional. Além disso, pretendia-se que a saúde suplementar estivesse alinhada aos princípios do SUS e comprometida com o alcance de melhores níveis de saúde pela população ^16^.

Elaborou-se uma política de qualificação dos planos de saúde que estimulava a adoção de programas de promoção da saúde e prevenção de doenças por meio de incentivos financeiros e assistenciais. Um exemplo foi a prorrogação dos prazos para constituição dos ativos garantidores da previsão de risco às operadoras que ofertassem esse tipo de programa a seus beneficiários (RN 94).

Houve também a publicação de manuais técnicos com informações sobre a implantação dessas ações, que orientavam as operadoras na definição do perfil demográfico e epidemiológico de seus beneficiários para a vigilância em saúde. Em 2008, 47% das operadoras contavam com programas de promoção da saúde e prevenção das doenças em ao menos uma área, a maioria direcionados a adultos e idosos ^16^.

Em 2006, foi instituído o Programa de Qualificação da Saúde Suplementar (RN 139) ^17^. Tratava-se da avaliação anual das operadoras com registro ativo na agência a partir dos indicadores que compunham o IDSS ^16^. Ressalta-se que metade dos indicadores correspondiam à qualidade assistencial e os demais às dimensões econômico-financeira, de estrutura, operação e satisfação dos usuários. Os resultados obtidos pelas operadoras passaram a ser divulgados com vistas à redução da assimetria de informações entre atores do setor ^17^ ^,^ ^18^.

A inclusão de práticas preventivas, de promoção da saúde e atendimentos multiprofissionais no rol, a formulação de diretrizes clínicas, o incentivo ao parto normal, a assistência à saúde bucal e à saúde mental foram todas iniciativas que visavam à atenção integral aos beneficiários. Outros destaques foram a ampliação da cobertura dos planos coletivos para acidentes de trabalho, procedimentos de saúde ocupacional e provisão pelas operadoras de todos os materiais e recursos necessários às internações domiciliares.

Para o enfrentamento das negativas de coberturas assistenciais, foi implementada a Notificação de Investigação Preliminar (NIP), um dispositivo prévio à abertura de processo administrativo que buscava induzir à reparação voluntária pelas operadoras, evitando punições administrativas ou judicialização.

#### 2010-2012: a saída dos sanitaristas da Dicol e o incremento na regulação assistencial

Na transição do segundo mandato de Luiz Inácio Lula da Silva para o de Dilma Rousseff, houve fortalecimento do Partido do Movimento Democrático Brasileiro (PMDB) na coalizão de partidos governistas. Isso implicou mudanças na composição da Dicol com a saída de sanitaristas e indicações com perfil diversificado. Entre elas, a de Maurício Ceschin, ex-dirigente da Qualicorp e designado diretor-presidente da Dipro ^12^.

Apesar de a diretoria colegiada contar com integrantes ligados ao setor regulado, antes ou depois de ocuparem o cargo, mantiveram-se e expandiram-se as estratégias de proteção aos consumidores por meio da regulação assistencial. Isso pode ser atribuído, em parte, à consolidação do arcabouço institucional e à profissionalização da agência pela atuação de servidores admitidos por concurso público, no período anterior, com formação em Saúde Coletiva.

Quanto às iniciativas de regulação assistencial do período, destacam-se o Programa de Monitoramento da Qualidade Assistencial, os regimes de direção técnica, a constituição do *pool* de risco para os planos coletivos, a expansão da portabilidade de carências, o monitoramento das garantias de acesso e a determinação de prazos máximos de atendimento para consultas básicas (sete dias úteis) e especializadas (entre 10 e 14 dias úteis). O descumprimento destas últimas previa a suspensão da comercialização de novos planos pelas empresas até que se readequassem ^19^ ^,^ ^20^.

Em 2012, 301 planos de saúde de 38 operadoras tiveram suas vendas suspensas. As 16 operadoras reincidentes no descumprimento de normas ao longo dos quatro ciclos de monitoramento foram indicadas para o Regime de Direção Técnica, e as demais assinaram termos de compromisso para redução das reclamações junto à ANS ^21^.

A RN 264 ^22^, de 2011, consolidou o conceito de promoção da saúde e prevenção de riscos e doenças adotado para a saúde suplementar, além de estabelecer o formato dos programas e os incentivos à adesão de operadoras e beneficiários. A partir dessa norma, ficaram definidas três linhas gerais para a estruturação dos programas: promoção do envelhecimento ativo, iniciativas para grupos com características específicas (segundo faixa etária, ciclo de vida ou fator de risco determinado) e para pessoas que convivem com doenças crônico-degenerativas.

Quanto às vantagens para adesão aos programas de promoção da saúde, a norma contempla os beneficiários com bonificações e prêmios regulamentados pela RN 265 ^23^. Para as operadoras, os benefícios são o aproveitamento dos investimentos realizados nesses programas para a redução da margem financeira de solvência exigida e o recebimento de pontuação extra no IDSS ^23^.

#### 2013-2014: mudanças na configuração da Dicol e o aprimoramento de medidas anteriores de regulação assistencial

Esse período foi marcado por mudanças frequentes na configuração da Dicol, predomínio de dirigentes ligados ao setor regulado e pela curta passagem de André Longo, médico vinculado ao sindicato de sua categoria em Pernambuco, como diretor e presidente da Dipro. O mandato mais curto foi consequência de sua atuação por um ano como dirigente da Dides e sua indicação pode ser compreendida como uma aproximação com a categoria médica ^12^.

Essa gestão teve como marca a continuidade de medidas anteriores para a regulação assistencial, a inclusão de 37 medicamentos orais para tratamento domiciliar do câncer e oito medicamentos contra efeitos colaterais no rol de janeiro de 2014 (RN 349), além do estímulo ao parto natural por meio do programa Parto Adequado. Ressalta-se que, em 18 meses, a taxa de partos normais entre os 35 hospitais participantes do programa teve um crescimento médio de 43%. Ademais, segundo estimativas, teriam sido evitadas aproximadamente 10 mil cesáreas sem indicação clínica e 400 admissões de bebês em unidade de terapia intensiva (UTI) neonatal ^24^.

#### 2015-2018: novo arranjo político-institucional e ampliação da participação social na atualização do rol

Nesse período, a medida com maior impacto para a saúde suplementar aconteceu fora da ANS. A *Lei nº 13.097* ^25^, de 2015, permitiu a participação de capital estrangeiro nos setores privado e filantrópico de assistência à saúde, colaborando com o processo de financeirização e concentração do setor por meio de fusões, aquisições e abertura do capital das empresas de planos de saúde na bolsa de valores ^26^ ^,^ ^27^ ^,^ ^28^.

A ampliação da influência do Movimento Democrático Brasileiro (MDB) e do empresariado do setor para composição da Dicol se expressou na indicação de José Carlos Abrahão, ex-presidente da Confederação Nacional de Saúde, Hospitais, Estabelecimentos e Serviços (CNSaúde) para a função de diretor-presidente em 2015. Concomitantemente, servidoras de carreira substituíram os sanitaristas historicamente ligados ao PT na direção da Difis e Dides. Assim, o jogo político na ANS passou a ser protagonizado por liberais ligados às empresas do setor e aos servidores ^12^.

Apesar da nova composição da Dicol, não houve mudanças expressivas no direcionamento das ações de regulação assistencial. Foram aperfeiçoadas ou tiveram continuidade medidas que visavam à avaliação de desempenho e à qualificação das empresas e dos prestadores para a transformação do modelo assistencial. Isso se expressou no Programa de Qualificação das Operadoras (PQO) regulamentado pela RN 386, que tem no IDSS a metodologia para avaliação com indicadores organizados em quatro dimensões: qualidade da atenção à saúde (IDQS), garantia de acessos (IDGA), sustentabilidade no mercado e gestão de processos e regulação ^29^.

Os dados para cálculo do IDSS são obtidos a partir dos sistemas de informação da ANS e, desde 2017, o padrão de Trocas de Informações na Saúde Suplementar (TISS) é utilizado para aprimorar a qualidade da informação utilizada na avaliação de desempenho das operadoras ^29^. Os indicadores que compõem as dimensões da qualidade da assistência à saúde e da garantia de acesso abordam a promoção da saúde e prevenção de doenças, o acesso aos profissionais e a oferta de serviços das redes assistenciais dos planos de saúde.

Os resultados descritos pelo IDSS-TISS para o ano base de 2017 demonstram uma concentração das maiores notas entre as operadoras de médio e grande porte. As operadoras que apresentam as menores notas são aquelas com menos beneficiários, apesar de representarem parcela expressiva das operadoras do setor ^29^, conforme observa-se na Tabela 2.

**Tabela 2:** Índice de Desempenho da Saúde Suplementar (IDSS) por porte da operadora de planos de saúde para o ano base de 2017.

Porte (beneficiários)	Total de beneficiários Ano base 2017	OPS Ano base 2017	IDSS Ano base 2017
Pequeno (1 a 19.999)	4.392.233	617	0,5727
Médio (20.000 a 99.999)	13.726.038	297	0,6389
Grande (mais que 100.000)	51.035.929	297	0,7673
Total geral	69.154.201	1.008	0,7295

OPS: operadoras de planos de saúde.

Fonte: Agência Nacional de Saúde Suplementar ^29^.

Há correlação positiva entre o maior porte das operadoras médico-hospitalares e seu desempenho superior no IDSS-TISS, o que pode refletir na sua melhor adaptação às exigências regulatórias. Contudo, destaca-se que as maiores empresas apresentarem notas altas de avaliação para os indicadores da dimensão de qualidade assistencial; porém, baixo desempenho na dimensão de garantia do acesso a profissionais de saúde, hospitais e laboratórios, como pode ser visto na Tabela 3.

**Tabela 3:** Índice de Desempenho da Saúde Suplementar (IDSS) das dez maiores operadoras de planos de saúde para o ano base de 2017.

Registro na ANS	Razão social	Modalidade	Média de beneficiários	IDSS	IDQS	IDGA
326305	Amil Assistência Médica Internacional S.A.	Medicina de grupo	5.645.902	0,9027	0,9940	0,7270
005711	BRADESCO Saúde S.A.	Seguradora especializada em saúde	3.476.511	0,9614	0,9381	0,4961
368253	Hapvida Assistencia Médica Ltda.	Medicina de grupo	3.446.529	0,5910	0,5970	0,2064
359017	Notre Dame Intermédica Saúde S.A.	Medicina de grupo	3.371.866	0,8698	0,8471	0,7546
006246	Sul América Companhia de Seguro Saúde	Seguradora especializada em saúde	2.149.453	0,7437	0,8433	0,3648
343889	Unimed Belo Horizonte Cooperativa de Trabalho Médico	Cooperativa médica	1.500.000	1,0000	1,0000	0,6260
339679	Central Nacional Unimed - Cooperativa Central	Cooperativa médica	1.486.494	0,9576	0,8258	0,6005
000582	Porto Seguro - Seguro Saúde S.A.	Seguradora especializada em saúde	733.935	0,7674	0,9843	0,3915
352501	Unimed Porto Alegre - Cooperativa Médica Ltda.	Cooperativa médica	711.168	0,9181	0,8665	0,3799
319996	Unimed do Estado de São Paulo - Federação Estadual das Cooperativas Médicas	Cooperativa médica	601.248	0,5458	0,4629	0,5406

ANS: Agência Nacional de Saúde Suplementar; IDGA: Índice de Desempenho de Garantia de Acessos; IDQS: índice de Desempenho de Qualidade da Atenção à Saúde (IDQS).

Fonte: Agência Nacional de Saúde Suplementar ^29^.

Outra ação de qualificação das empresas de planos de saúde é o Programa de Acreditação das Operadoras, cuja adesão implica incremento na pontuação do IDSS-TISS. Salienta-se que há correlação positiva entre operadoras acreditadas e melhor desempenho em 2017. Assim, 32 das 38 operadoras acreditadas situaram-se na faixa de pontuação alta para o IDSS (0,8-1,0) ^19^.

Apesar de a *Lei nº 9.961* ^9^, de 2000, estabelecer a ANS como reguladora das relações entre operadoras, beneficiários e prestadores de serviços, ela dispõe de poucos mecanismos para atuar junto aos últimos. Assim, para qualificar a assistência e aumentar o nível de informação para a escolha dos prestadores pelas operadoras e beneficiários, foi estabelecido o Programa de Divulgação da Qualificação de Prestadores de Serviços na Saúde Suplementar ^30^. A adesão dos prestadores a esse programa é voluntária e o principal estímulo é a utilização dos certificados de acreditação ou de qualidade monitorada como parte do fator de qualidade aplicado ao índice de reajuste estipulado pela ANS ^31^.

A proibição, pela *Lei nº 9.656* ^8^, de 1998, da venda de planos de saúde fora das segmentações definidas foi regulamentada por normas posteriores que detalharam questões como: as circunstâncias nas quais os procedimentos de urgência e emergência deverão ser cobertos conforme a segmentação do plano de saúde contratado ^32^, a obrigatoriedade de oferta do plano de referência a todos os consumidores, atuais ou futuros ^33^ (exceção feita às autogestões e operadoras exclusivamente odontológicas) e a oferta de serviços voltados para a saúde ocupacional e não previstos no rol de procedimentos ^34^.

Todavia, a subsegmentação das coberturas assistenciais é uma pauta das empresas de planos de saúde e suas entidades representativas, sobretudo nos momentos de crises do setor. Na década de 2000, houve articulação política e pressão do empresariado sobre órgãos do Governo Federal para a oferta de coberturas mais restritivas, entretanto, as propostas não prosperaram.

Outra tentativa ocorreu em 2016 com a chegada de Ricardo Barros ao Ministério da Saúde e seu projeto de Plano de Saúde Acessível. O ministro articulou um Grupo de Trabalho com a participação de representantes do Ministério da Saúde, ANS e da Confederação Nacional das Empresas de Seguros Gerais, Previdência Privada, Saúde Suplementar e Capitalização (CNSEG) ^35^ ^,^ ^36^. A ideia seria propor planos de saúde que ofertassem um conjunto de ações mais restrito em relação àquelas exigidas pela ANS e que, supostamente, seria mais barato e acessível às famílias de menor renda. Ao final das discussões desse grupo, solicitou-se à ANS uma análise de viabilidade técnica para implementação de três possíveis subsegmentações: plano simplificado, plano ambulatorial+hospitalar e plano em regime misto de pagamento.

O projeto de planos acessíveis passou pela avaliação técnica de servidores da ANS e por uma etapa de participação social que contou com outros sujeitos do processo regulatório. Ao final desse processo, indicou-se a rejeição da proposta pelo fato de as subsegmentações elaboradas já estarem contempladas nas ações regulatórias existentes ^35^.

Em agosto de 2018, Rogério Scarabel Barbosa assumiu o cargo de diretor presidente da ANS por indicação do presidente Michel Temer. Essa nomeação foi cercada de polêmicas devido à sua atuação pregressa como advogado da administradora de benefícios Hapvida. Em setembro do mesmo ano, Paulo Rebello Filho, ex-chefe de gabinete do Ministro da Saúde Ricardo Barros, assumiu a direção da Diope. Nesse período, as demais diretorias da ANS seguiram ocupadas por servidoras de carreira.

A regulação assistencial no período foi caracterizada pelo monitoramento dos resultados e continuidade de projetos, como a segunda fase de Parto Adequado, Idoso Bem Cuidado e Promoção da Saúde e Prevenção de Riscos e Doenças, além da inclusão de novos itens ao rol de procedimentos ^37^. Outra iniciativa importante foi a extensão das regras de portabilidade de carências para os beneficiários de planos de saúde coletivos empresariais a partir de junho de 2019 ^11^.

Ademais, foram realizadas mudanças no ciclo para a elaboração do rol para aumentar a participação da sociedade na sua atualização. Assim, em 2018, disponibilizou-se por tempo determinado no site da ANS um formulário (Formrol) específico para que qualquer pessoa física ou jurídica sugerisse propostas de atualização do rol (PAR). As PAR podem ser empregadas para a solicitação de incorporação ou desincorporação de tecnologia em saúde, inclusão, exclusão ou alteração de Diretriz de Utilização (DUT), ou alteração de termo descritivo de procedimento, ou evento em saúde já listado no rol. Salienta-se que o envio de informações e documentos técnicos é pré-requisito para o recebimento e a análise das sugestões ^38^.

Anteriormente, apenas os membros do Comitê Permanente de Regulação da Atenção à Saúde (Cosaúde) poderiam encaminhar tais propostas. Com a mudança, a participação social na elaboração do rol deixou de ser restrita à etapa de consulta pública que antecedia sua publicação ^11^. Diferentemente dos mecanismos de participação social no SUS, a possibilidade de participar da construção desse aspecto da política de saúde suplementar é mediada por critérios técnicos, o que pode implicar em barreiras à participação de sujeitos que não contam com a expertise necessária à elaboração dos documentos e pré-requisitos solicitados.

Em 2021, a recepção das PAR passou a ser contínua e não mais no início do ciclo de atualização semestral do rol ^38^. No ano seguinte, com a promulgação da *Lei nº 14.307* e sua regulamentação pela RN 555, a atualização do rol passou por novas mudanças. Foi restabelecida a Comissão de Atualização do Rol de Procedimentos e Eventos em Saúde Suplementar e instituído o prazo de 180 dias (prorrogáveis por outros 90 dias) para a finalização do processo administrativo de incorporação de tecnologias. Ainda, a avaliação da incorporação de tratamentos antineoplásicos de uso oral se tornou prioritária com o prazo de 120 dias (prorrogáveis por outros 60 dias) para definição sobre a solicitação. Após isso, o fornecimento do medicamento ao beneficiário, ou ao seu representante legal, deverá acontecer em até dez dias após a prescrição médica.

Embora a periodicidade anterior de dois anos para a atualização do rol não acompanhasse o ritmo das inovações tecnológicas em saúde, o prazo de seis meses pode ser insuficiente para a adequada avaliação técnica e administrativa da incorporação de tecnologias assistenciais.

As reclamações sobre o descumprimento do rol ainda são as mais frequentes no âmbito da regulação assistencial ^11^. Isso reflete a persistência da prática abusiva de negação de coberturas pelas empresas em detrimento da regulação vigente.

A ANS conta com mecanismos administrativos e de fiscalização para o monitoramento e o acompanhamento da garantia de coberturas assistenciais pelas operadoras. A agência pode realizar visita técnico-assistencial para verificar anormalidades, suspender a venda dos planos ofertados pelas operadoras e, nos casos mais graves, instituir um Plano de Recuperação Assistencial (PRASS) com duração de até 12 meses. Para as operadoras que não conseguem se recuperar nesse prazo, é implantado um Regime Especial de Direção Técnica, em que a ANS nomeia um diretor para atuar na gestão da empresa com proventos pagos por ela. Caso a recuperação não seja possível, sua saída do mercado acontece sob supervisão da agência, e sua carteira de beneficiários é transferida para outra operadora.

O mapeamento do risco assistencial nas operadoras de planos de saúde é realizado trimestralmente a partir de informações coletadas nos sistemas de informações da ANS ^39^. Porém, medidas administrativas podem ser adotadas pela agência nas situações de risco assistencial independentemente desse monitoramento.

As iniciativas descritas demonstram o movimento da ANS em articular a proposição de um modelo integral de atenção à saúde e a racionalização dos custos assistenciais com efeitos benéficos para a saúde dos beneficiários de planos e para a sustentabilidade do setor. Todavia, há questões inerentes à assistência médica privada e características próprias ao modelo brasileiro de saúde suplementar que se colocam como desafios adicionais. Entre elas, destacam-se as formas de contratação e credenciamento de prestadores de serviços de saúde, que dificultam o estabelecimento de diretrizes clínico-preventivas uniformes e orientadas por evidências científicas, além do modelo de remuneração baseado no pagamento por procedimento realizado (*fee for service*). Porém, o estímulo à adoção de programas de promoção da saúde e prevenção de doenças e a avaliação da ANS sobre o desempenho e qualidade do setor são apostas interessantes para maior convergência entre o interesse público e privado na saúde suplementar.

O Quadro 1 resume as prioridades das diferentes gestões da ANS e a evolução dos seus componentes de regulação assistencial.

**Quadro 1:** Atuação da Agência Nacional de Saúde Suplementar (ANS) por gestão de diretores presidentes, de 2000 a 2018.

PERÍODO	DIRETOR-PRESIDENTE	CONTEXTO	ÁREAS PRIORITÁRIAS/AGENDA/LINHAS DE AÇÃO/DECISÕES-CHAVE	REGULAÇÃO ASSISTENCIAL - COMPONENTES E EVOLUÇÃO
2000-2003	Januário Montone	2º Governo Fernando Henrique Cardoso; Influência do Ministro da Saúde José Serra nas definições sobre a agência e indicações para três diretorias (gestores do governo - duas do Ministério da Saúde e uma do Ministério da Fazenda); Diretora vinculada aos interesses dos consumidores (Difis).	Implantação e organização interna da agência; Ênfase na regulação econômico-financeira (a partir da experiência da Susep); Acompanhamento dos efeitos da regulação econômico-financeira no mercado.	Responsável pela consolidação do rol de procedimentos e eventos em saúde; Definição de bases regulatórias: tipos de segmentação dos planos, modelos de contratos; bases/sistemas de informações.
2004-2006	Fausto Pereira dos Santos	1º governo do PT (Luiz Inácio Lula da Silva); Formação de coalizões para o governo - multiplicidade de atores; Apenas a direção da Difis não foi trocada na transição; Três indicações do PT (Dipro, Diges e Dides); Diope - Indicação da Amil (Apoiada pelo PTB - base aliada); Fortalecimento dos grupos sanitaristas na ANS.	Protagonismo da regulação assistencial; Mudança no modelo assistencial da saúde suplementar a partir do fortalecimento das ideias de prevenção e promoção da saúde; Incentivos econômicos e assistenciais às operadoras; Aproximação com princípios do SUS; Portabilidade de carências - Regulamentação da mudança entre planos individuais pelos clientes; 1º Concurso público para a ANS (2005).	Programa de Qualificação da Saúde Suplementar (RN 139); IDSS; RN 94 - Extensão dos prazos para configuração de reservas financeiras por operacionalização de programas de prevenção e promoção da saúde; Inclusão de práticas preventivas e de promoção da saúde no rol de procedimentos e incorporação de equipes multiprofissionais na assistência à saúde dos clientes.
2007-2009	Fausto Pereira dos Santos	2º governo do PT (Luiz Inácio Lula Silva); 3 dos 5 diretores foram reconduzidos (Dipro, Dides e Diope); Mudanças na Diges e Difis; Fortalecimento dos sanitaristas nas diretorias; Consolidação da Diope como canal aos interesses do empresariado (recondução do diretor indicado pela Amil e PTB); Intensificação da concentração do mercado por meio de aquisições.	Iniciativas para regulação dos planos coletivos; Reajustes anuais dos planos coletivos. Regulamentação das administradoras de benefícios - RN 195 e RN 196; Ênfase menor na fiscalização; Fragilidades na integração com o SUS (ressarcimento); Implantação da Notificação de Intermediação Preliminar (NIP); 2º Concurso público para a ANS (2007).	Atualização do rol de procedimentos para inclusão das coberturas de saúde ocupacional e acidentes de trabalho.
2010-2012	Maurício Ceschin	Transição do 2º governo de Luiz Inácio Lula da Silva para o 1º mandato de Dilma Rousseff (2011); Maior destaque para o PMDB na coalizão de partidos governistas; Mudança na configuração política da ANS; Diretoria colegiada com perfil diversificado e mais liberal; Diretor-presidente/Dipro ligado ao mercado - ex-presidente da Qualicorp; Novo diretor da Diope com passagem pela Amil apoiado pelo PMDB-RJ; Diretor da Difis oriundo do ministério da fazenda; Aquisição da Amil pelo grupo United Health.	Intensificação da regulação assistencial; Iniciativas para compensar lacunas na regulação dos prestadores de serviços; Retificação de falhas assistenciais por meio do regime especial de direção técnica; Aprofundamento nas mudanças da portabilidade de carências; *Pool* de risco para enfrentamento da falsa coletivização e problemas na regulação dos reajustes de planos coletivos; Determinação dos prazos máximos para atendimento; Aperfeiçoamento do processo de ressarcimento ao SUS; Regulamentação da NIP; Implementação da Agenda Regulatória (2011-2012) - instrumento de planejamento visando previsibilidade e transparência às ações da ANS; RN 279/2011 - Inclusão de aposentados e demitidos em contratos específicos para cálculo de reajustes.	RN 267 - Programa de Incentivo à Qualificação de Prestadores de Serviços na Saúde Suplementar (Qualiss); Qualiss indicadores - monitoramento da atuação dos prestadores de serviços; RN 256 - regulamenta o regime de direção técnica das operadoras; Portabilidade para os beneficiários de planos coletivos por adesão; *Pool* de risco implicou no agrupamento de contratos de até 30 vidas para cálculo de reajuste; RN 259 - regulamentação dos prazos máximos para atendimento - medida de maior impacto para o setor; Melhoria na coleta e transmissão das informações para o ressarcimento ao SUS; Regulamentação da NIP: mediação de conflitos relativos às negativas de coberturas entre clientes e operadoras.
2013-2014	André Longo	1º mandato de Dilma Rousseff; Maior rotatividade dos diretores; Mandato mais curto do diretor-presidente (1 ano); Predomínio de diretores liberais e ligados ao mercado; Não houve redirecionamento no modelo regulatório; Gestão marcada pelo aprimoramento de iniciativas anteriores; *Lei nº 13.003/2014* - torna obrigatória a existência de contratos escritos entre operadoras e prestadores de serviços.	Continuidade de medidas anteriores para regulação assistencial; Incremento na arrecadação de multas e valores do ressarcimento ao SUS (medidas gerenciais e parcelamentos); RN 323 - Criação de ouvidorias pelas operadoras; Operacionalização do ressarcimento de exames e terapias ambulatoriais de média e alta complexidade por meio da Autorização de Procedimento Ambulatorial (APAC); Estímulo ao parto normal.	RN 349 - inclusão no rol de medicamentos para tratamento domiciliar do câncer e de efeitos colaterais RN 368 - acesso à informação das beneficiárias aos percentuais de cirurgias cesáreas e de partos normais, por operadora, por estabelecimento de saúde e por médico e utilização do partograma, cartão da gestante e carta de informação à gestante; Projeto Parto Adequado - parceria entre ANS, Hospital Einstein e Instituto de Melhoria dos Cuidados de Saúde (IHI, acrônimo em inglês) dos Estados Unidos, com apoio do Ministério da Saúde, para promoção do parto normal, qualificação dos serviços da saúde suplementar e redução de cesáreas desnecessárias.
2015-2017	José Carlos Abrahão (até maio de 2017) Leandro Fonseca da Silva (maio de 2017 - agosto de 2018)	2º mandato de Dilma Rousseff (2015); *Lei n° 13.097* (2015) - Abertura ao capital estrangeiro na oferta de serviços de saúde; Disputa entre PMDB e PT pela indicação dos diretores; Fortalecimento do PMDB (Senado) nas definições sobre a composição da diretoria da ANS; Diretor-presidente/Dipro e diretor da Diope liberais e ligados ao mercado; Ascensão às diretorias de servidores concursados em substituição aos sanitaristas ligados ao PT nas demais diretorias.	Monitoramento de resultados e aprimoramento de iniciativas anteriores. Ex: Projeto Parto Adequado e Programa de Qualificação das Operadoras; Projeto registro eletrônico em saúde; Dificuldades para operacionalizar o acesso ao cartão do SUS para 100% dos clientes da saúde suplementar.	96,24% dos beneficiários em operadoras com IDSS superior a 60%; Eventos para incentivo à adoção de programas de promoção da saúde e prevenção de doenças na saúde suplementar; Incentivo à implementação de planos de cuidados do idoso e plano de cuidado nascer saudável.
2018	Rogério Scarabel Barbosa (2018 - 2021)	Diretor presidente/Dipro e Diope liberais e ligados ao mercado;Demais diretorias ocupadas por servidores concursados da ANS; Consolidação do padrão de distribuição entre os dois grupos nas diretorias da agência; Não há nenhum profissional de saúde entre os diretores da ANS; Suspensão pelo STF da resolução da ANS sobre franquia e coparticipação - Aumento da coparticipação para 40% ou 60%, a depender do contrato, fixação de limites mensais e anuais elevados para o pagamento dessas modalidades, cobrança em pronto atendimento.	RN 439 - nova metodologia sobre o processo de atualização do rol de procedimentos e eventos em saúde;RN 438 - ampliação das regras de portabilidade de carências para os beneficiários de planos de saúde coletivos empresariais (válida a partir de junho de 2019).	Ampliação da participação social durante os ciclos de atualização do rol - formulário específico (FormRol) disponibilizado no portal da ANS no início dos ciclos de atualização.

Dides: Diretoria de Desenvolvimento Setorial; Difis: Diretoria de Fiscalização; Diges: Diretoria de Gestão; Diope: Diretoria de Normas e Habilitação das Operadoras; Dipro: Diretoria de Normas e Habilitação de Produtos; IDSS: Índice de Desempenho da Saúde Suplementar; NIP: Notificação de Investigação Preliminar; PMDB: Partido do Movimento Democrático Brasileiro; PMDB-RJ: Partido do Movimento Democrático Brasileiro do Rio de Janeiro; PT: Partido dos Trabalhadores; PTB: Partido Trabalhista Brasileiro; RN: Resolução Normativa; STF: Supremo Tribunal Federal; SUS: Sistema Único de Saúde; Susep: Superintendência de Seguros Privados.

Fonte: elaboração própria.

## Conclusão

Este estudo mostrou não apenas diferenças de ênfase nas estratégias de regulação assistencial ao longo das gestões da ANS, mas também elementos de continuidade e mudanças incrementais em iniciativas regulatórias, apesar das mudanças político-partidárias dos governos, suas coalizões e as pressões por desregulamentação do empresariado e suas entidades representativas. Isso pode ser atribuído, em parte, à atuação de alguns diretores da ANS, às resistências dos servidores públicos, à profissionalização da regulação e à consolidação de um arcabouço institucional que considera os princípios do SUS. A presença crescente de servidores de carreira entre os dirigentes da ANS indica a importância que têm conquistado nas definições sobre as políticas regulatórias. Contudo, persistem pressões sobre a agência e seus servidores para a flexibilização da regulação assistencial e as intensas disputas entre grupos de interesse.

Ressalta-se a existência de lacunas regulatórias que limitam a atuação da ANS em temas estruturantes do modelo assistencial da saúde suplementar, tais como a ausência de mecanismos para a regulação das atividades dos prestadores de serviços. Entretanto, a crescente verticalização das redes assistenciais por operadoras e hospitais privados, que também passaram a ofertar planos de saúde, apresenta novas questões para a regulação assistencial, demandando reavaliações dos instrumentos vigentes para a promoção do interesse público e o equilíbrio do setor. Ademais, deve-se atentar à permeabilidade da ANS ao empresariado, sob o risco de captura das ações da agência por seus interesses.

Neste estudo, não foi possível aprofundar análises sobre os processos políticos que envolvem a regulação da saúde suplementar e sua correlação de forças, pois a crise associada à pandemia de COVID-19 dificultou a realização de entrevistas com atores estatais e não-estatais. Assim, pesquisas adicionais capazes de caracterizar os grupos sociais envolvidos nas disputas pela direcionalidade da política regulatória e de analisar suas ações, suas estratégias, seus deslocamentos políticos e suas contradições fundamentais são necessárias.

Outras limitações, correspondem à análise das correlações entre o IDSS e a concentração do setor de saúde suplementar, e a correlação entre as notas altas de avaliação para os indicadores da dimensão de qualidade assistencial; porém, houve baixo desempenho na dimensão de garantia do acesso a profissionais de saúde, hospitais e laboratórios apresentadas pelas operadoras de maior porte verificadas em 2017. Sugere-se que estudos com ênfase nesses componentes da avaliação de desempenho das operadoras sejam realizados.

O estudo sugere a necessidade de aprimoramento do marco regulatório da saúde suplementar, de forma participativa e democrática, visando possibilitar que diferentes atores influenciem a construção do modelo regulatório. Entretanto, os interesses empresariais dificultam a implementação de mudanças que favoreçam a proteção aos beneficiários. Mais do que isso, o fortalecimento e o dinamismo do setor privado na saúde no país nas últimas décadas, sob incentivo do Estado, expõem conflitos distributivos que dificultam a consolidação do SUS.
